# Supplementary material for: TastepepAI: An artificial intelligence platform for taste peptide de novo design
Source: PLoS Comput Biol. 2025 Oct 16;21(10):e1013602. doi: 10.1371/journal.pcbi.1013602 (PMC12543283; doi:10.1371/journal.pcbi.1013602)
Supplement: S1 File — (ZIP) [file pcbi.1013602.s006.zip › Supplementary Data 2/TaPep10 HPLC.doc]

**HPLC REPORT**

| Sample: | FCL-NJP93311 TaPep10 YP-6 | | | Analyzed date: | 2024-9-23 |
| --- | --- | --- | --- | --- | --- |
| Analyst: | WJ | | | Reconstitution: | 100%H_2_O |
| Lot. No.: | NJP93311-1172227 | | | | |
| Column: | 4.6×250mm,Venusil MP C18-5 | | | | |
| Solvent A | A: 0.1% Trifluoroacetic Acid in 100% Acetonitrile | | | | |
| Solvent B | B: 0.1% Trifluoroacetic Acid in 100% Water | | | | |
| Gradient: |  | A | B |  | |
|  | 0.0min | 5% | 95% |  | |
|  | 25.0min | 30% | 70% |  | |
|  | 25.1min | 100% | 0% |  | |
|  | 30.0min | Stop | |  | |
| Volume: | 5μl | | |  | |
| Wavelength: | 220nm | | |  | |
| Flow rate: | 1.0ml/min | | |  | |

─────────────────────────────────────────────────────────────

Rank	Time	Conc. Area	Height

─────────────────────────────────────────────────────────────

1	4.963	0.4031	6917	770

2	5.577	0.6510	11170	981

3	6.113	0.8185	14044	1869

4	6.329	96.7620	1660358	151956

5	6.857	0.3844	6596	830

6	7.119	0.9810	16833	1673

─────────────────────────────────────────────────────────────

Total	100	1715918	158079
